# Stoichiometric analysis of 20 amino acids, thermogravimetric parameters and trace elements in five types of tea from Guizhou, China, based on entropy analysis (EA) and factor cluster analysis (FCA)

**DOI:** 10.1016/j.fochx.2025.102457

**Published:** 2025-04-14

**Authors:** Libing Zhou, Chunli Huang

**Affiliations:** Guangxi Science & Technology Normal University, Laibin 546199, Guangxi, China

**Keywords:** Tea, Thermogravimetric analysis, ICP–OES, Trace elements, Amino acids

## Abstract

This study employed a range of indicators, including heat of combustion, combustibility, fat content, amino acid content, ash content, crude fiber content, trace element content, and concentrations of catechins, theanine, and caffeine, to develop comprehensive evaluation systems for assessing the quality of five tea samples: Duyun Maojian tea, Liping fragrant tea, Shuicheng spring tea, Huaxi jasmine tea, and Zunyi black tea. Analytical techniques such as gray pattern recognition (GPR), gray principal component analysis (GPCA), gray factor analysis (GFA), entropy analysis (EA), and factor cluster analysis (FCA) were utilized. Based on the 42variable data, the nutritional value ranked as follows: Shuicheng spring tea > Huaxi jasmine tea > Duyun Maojian tea > Liping fragrant tea > Zunyi black tea. These findings offer significant theoretical and practical insights for the determination and comprehensive evaluation of tea quality indicators, contributing to scientific research on the nutrition, health, and classification aspects of tea.

## Highlights

• Stoichiometric analysis of 20 amino acids, trace elements, etc., in 5 teas.
• Thermogravimetric analysis was used for food combustion stability evaluation.
• Construction of food comprehensive evaluation systems.
• This study provides a new idea for food nutrition evaluation.

## 1 Introduction

Tea is considered to be among the world's oldest and most widely consumed beverages, playing a significant cultural and social role, particularly in China, where its origins date back to millennia. Among the various tea-producing regions in China, Guizhou Province is notable for its unique geographic features and favorable climate, which have facilitated the cultivation of various tea varieties (Ma et al., 2024).Duyun Maojian tea is a green tea produced in Duyun, Guizhou Province, China. Tea buds are renowned for their tenderness and fresh flavor. Duyun Mao Jian Tea is distinguished by its high fragrance and fresh taste and is frequently regarded as one of the most exquisite varieties of green tea. Tea is rich in polyphenols and has been demonstrated to have antioxidant, hypoglycemic, hypolipidemic, and anti-inflammatory effects(M. L. Zhang et al., 2020; Zhang, Casasent, et al., 2024). Liping fragrant tea, which has a provenance in Liping County in Guizhou Province, China, is a distinguished premium tea known for its robust fresh aroma, crisp flavor, and tender green leaves (Ling, 2017). The favorable climate and geography of Liping County provide an optimal environment for tea cultivation, which in turn contributes to the high-quality and esteemed reputation of Guizhou Liping fragrant tea. Shuicheng spring tea enhances immune function, lowers blood pressure, and eliminates fatigue (Donglian et al., 2021). Huaxi jasmine tea is effective in sterilization, bacteriostasis, prevention of cardiovascular disease, and enhancement of immunity (Maiquan et al., 2022). Zunyi black tea possesses immunoregulatory, anti-inflammatory, and antioxidant properties. Specific compounds that contribute to the distinctive sweet aroma of tea have been identified, including geraniol, 2-phenylethyl alcohol, and cinnamaldehyde. Furthermore, the interactions between these compounds and their receptors on the nose were investigated. These findings revealed that these compounds bind to specific receptors on the nose, thereby eliciting the perception of a pleasant aroma. By elucidating this process, it may be possible to further enhance the aroma of Zunyi black tea(Sun et al., 2024).

The consumption of tea, one of the most popular non-alcoholic beverages worldwide, is often associated with various health benefits. However, in addition to its nutrient composition, tea may contain non-essential trace elements that can have toxic effects. As food components undergo biotransformation in the gastrointestinal tract after ingestion, it is crucial to evaluate both the total and bioavailable contents of these trace elements. This study aimed to provide comprehensive data on the influence of in vitro digestion of 16 trace elements present in ready-to-drink iced teas, including black, green, mate, and white teas. The concentrations of essential minerals (Co, Cr, Cu, Fe, Mn, Se, and Zn) and inorganic impurities (Al, As, Cd, Li, Ni, Pb, Sb, Sn, and Sr) were determined via ICP–OES after microwave acid digestion. Bioaccessibility was assessed by simulating gastric (pepsin) and intestinal juices (pancreatin and bile salts), and bioavailability was evaluated using Caco-2 cell culture as an intestinal epithelial model. Tannins were also evaluated using UV-VIS spectroscopy. Multivariate analysis allowed the classification of the iced tea samples into three groups based on their trace element profiles. The bioaccessible fractions of Al, Cu, Sr, Mn, and Zn corresponded to approximately 40–60 % of their total content. The bioaccessibility and bioavailability of Mn exhibited similar patterns, with green iced tea showing higher values than black iced tea and mate-iced tea. Additionally, the bioavailability of Sr in green tea was found to be 50 % greater than that in black tea (Milani et al., 2020).

In summary, sensory evaluation of tea has been dominant in research on multiple indicators of tea nutrition and quality. With the development of science and technology, the research scope of multi-indicator analysis of tea has expanded from sensory evaluation to tea inclusion, physicochemical indicators, and other aspects. To better protect and develop the local tea industry in Guizhou, it is particularly important to study the multiple components and quality characteristics of the tea. Specifically, the analysis of amino acids, thermogravimetric parameters, trace elements, fats, and other indicators can not only reveal the nutritional value and sensory characteristics of tea but also provide a scientific basis for its production and consumption.

Therefore, it is crucial to assess tea quality using a variety of indicators. By analyzing factors such as nutrient content, bioactivity, the presence of trace elements, and potential health effects, a more comprehensive understanding of tea quality can be achieved. This multifaceted approach can provide valuable insights into the overall quality and potential benefits of different types of tea.

This paper focuses on five varieties of tea, namely, Duyun Maojian tea, Liping fragrant tea, Shuicheng spring tea, Huaxi jasmine tea and Zunyi black tea, all of which originate from Guizhou Province. Determination of the heat of combustion, combustion stability, fat content, crude fiber content, ash content, amino acid content, trace elements, catechins, theanine, and caffeine content provides a comprehensive understanding of these teas. The establishment of a multi-index analysis and evaluation system for these teas provides a robust scientific foundation for the large-scale development of tea resources and classification of tea. The findings of this research have the potential to make a significant contribution to the field of tea studies and enhance our understanding of the distinctive characteristics of Guizhou teas.

## 2 Materials and methods

### 2.1 Materials and instruments

Five types of tea, Duyun Maojian tea, Liping fragrant tea, Shuicheng spring tea, Huaxi jasmine tea and Zunyi black tea, from Guizhou Province in China were selected as analytical samples and were identified by Prof. Caiyun Jiang of Guangxi Science & Technology Normal University and stored at Guangxi Science & Technology Normal University. The samples were finely ground in a mortar and passed through a 40-mesh pharmacopoeia sieve. Duyun Maojian tea (produced in May 2020) originated in Maojian town, Duyun city, Qiannan Buyei and Miao Autonomous Prefecture, Guizhou Province; Liping fragrant tea (produced in March 2020) originated in Liping County, Qiandongnan Miao and Dong Autonomous Prefecture, Guizhou Province; Shuicheng spring tea (produced in March 2020) originated in Shuicheng County, Liupan city, Guizhou Province; Huaxi jasmine tea (produced in May 2020) originated in Huaxi District, Guiyang city, Guizhou Province; and Zunyi black tea (produced in February 2021) originated in Meitan County, Zunyi city, Guizhou Province.

The equipment used in this study was an HR-15BH series combustion calorimetry experimental apparatus; ignition wire (nickel‑chromium wire); tablet press (Changxing Higher Education Instrument Equipment Development Co., Ltd.); grinder (FW135, Taisite Instrument Co., Ltd., Tianjin); FA200 electronic balance (Shanghai Shunyu Hengping Scientific Instrument Co., Ltd.); NETZSCH STA2500 thermogravimetric analyzer, crucibles (Germany NETZSCH Company); SE206 fat analyzer; F1600 fully automatic fiber analyzer (Jinan Alva Instrument Co., Ltd.); AUY120 microanalytical balance (Shimadzu Corporation); GZX-GF101–3-S drying oven (Shanghai Huyue Ming Science Instrument Co., Ltd.); SX-4-10P muffle furnace (Taisite Instrument Co., Ltd., Tianjin); ICP-OES spectrometer (iCAP 7000 SERIES, Thermo Scientific, USA); A300 automatic amino acid analyzer (Germany Manmboer Company); and Waters-2695 HPLC (Waters Corporation, USA).

The main chemical reagents used in this study were benzoic acid (AR, Tianjin Comio Chemical Reagent Co., Ltd.); pharmaceutical capsules (Guangdong Biological Co., Ltd.); high-purity oxygen; aluminum trioxide; 99.999 % high-purity argon; 30–60 °C boiling-range petroleum ether (AR, Chengdu Jinshan Chemical Reagent Co., Ltd.); potassium hydroxide (AR, Tianjin Damao Chemical Reagent Co., Ltd.); hydrochloric acid (AR, Xilong Science Co. Ltd.); sulfuric acid (AR, Xilong Science Co., Ltd.); multielement mixed liquid standard sample (GNM-M218741–2013); 30 % hydrogen peroxide (AR, Xilong Science Co., Ltd.); nitric acid (GR, Chengdu Jinshan Chemical Reagent Co., Ltd.); porcelain crucible; ninhydrin (AR, Shanghai Aladdin Biochemical Technology Co., Ltd.); amino acid mixed standard 650–0037 Feedstuff (Mann-Moble, Germany); sodium acetate trihydrate (AR, Xilong Science Co., Ltd.); formic acid (AR, Tianjin Opusheng Chemical Co., Ltd.); acetic acid (AR, Chengdu Kolon Chemical Co., Ltd.); and sodium hydroxide (AR, Xilong Science Co., Ltd.).

### 2.2 Measurement method for each index

The heat of combustion, fat content, ash content, thermogravimetric parameters, crude fiber content, and trace element content of the five teas were determined according to the literature (L. B. Zhou & Hou, 2024).

The catechin, theanine, and caffeine contents in the five tea samples were determined via high-performance liquid chromatography (HPLC) with reference to GB/T 8313–2018, GB/T 23193–2017 and GB 5009.139–2014, respectively. The HPLC chromatograms of the catechins, theanine, and caffeine in Duyun Maojian tea are shown in Figure sa1, Figure sa2 and Figure sa3.

The amino acid content was determined as follows: 0.4 g of sample was taken, the volume was adjusted to 50 ml with 12 mmol/L diluted hydrochloric acid, the sample was then filtered and passed through a 0.22 μm aqueous membrane, and 1 ml was removed from the injection bottle. The operational procedure of the A300 automatic amino acid analyzer has been previously described (Huojiao & Libing, 2024). Each sample analysis was repeated thrice (*n* = 3, RSD% < 2 %).

### 2.3 Multi-index comprehensive evaluation method

In this study, gray pattern recognition (GPR), gray principal component analysis (GPCA), gray factor analysis (GFA), entropy analysis (EA), and factor cluster analysis (FCA) were used to develop comprehensive evaluation systems for five types of tea, including multiple indicators of heat of combustion, combustibility (tea combustion stability), fat content, amino acids, ash, crude fiber, trace elements, catechins, theanine, and caffeine.

#### 2.3.1 Gray pattern recognition (GPR)

Gray pattern recognition, a prevalent methodology in gray system theory, serves as a pivotal technique for evaluating various schemes through gray metrology. This method leverages the correlation coefficients of the ideal scheme, which comprises optimal indicators, to determine the gray correlation degree. Subsequent ranking based on the magnitude of the correlation degrees facilitates comprehensive analysis and formulation of conclusions. This study utilized gray pattern recognition (GPR) and thermogravimetry to analyze the combustion stability of tea. By studying the combustion characteristic indexes at different heating rates through thermogravimetric analysis, the thermal properties of tea were determined. The principles and methods of gray pattern recognition (GPR)for calculating the F value adhered to those outlined in the literature previously published by the author (Libing Zhou, 2020).

The thermogravimetric parameters, including index X_1_, the first-stage weight loss percentage; index X_2_, the fastest temperature in the first stage of weight loss; index X_3_, the weight loss percentage in the second stage; index X_4_, the fastest temperature in the second stage of weight loss; index X_5_, the percentage of weight loss in the third stage; index X_6_, the remaining mass percentage; index X_7_, the peak area of the first stage; and index X_8_, the second-stage peak area, were used to assess tea combustion stability through gray pattern recognition. The thermal gravimetric parameter data of the 5 types of tea are shown in Table Sa1. Table Sa1 is provided in the supplementary material.

#### 2.3.2 Gray principal component analysis (GPCA)

Gray principal component analysis (GPCA) is used to analyze and process data in the field of gray systems theory. It is an extension of traditional principal component analysis (PCA), which is specifically designed to handle data with uncertainty and incompleteness. Gray principal component analysis is a method of studying samples by maximizing the retention of original information in the original sample set through appropriate mathematical transformations, based on the obtained gray correlation coefficient matrix, which is used as the principal component analysis correlation coefficient matrix for calculation, obtaining eigenvalues and eigenvectors, and calculating the variance and cumulative variance contribution rates of the gray principal component (Yang et al., 2013; Yeh et al., 2010).

#### 2.3.3 Gray factor analysis (GFA)

Gray factor analysis (GFA) is a statistical method employed for the analysis of data that encompasses both qualitative and quantitative information. It is particularly advantageous in circumstances where data are scarce or incomplete, and traditional statistical techniques are inapplicable (Colibazzi et al., 2008; Li et al., 2021). ^For gray factor analysis, the gray correlation coefficient of the sample was used to replace the sample correlation coefficient matrix in the factor analysis. The gray correlation coefficient matrix was used to participate in the computation and obtain the eigenvalues and eigenvectors, and the variance contribution rate and total variance contribution rate of the gray factor analysis were computed(^^Lin et al., 2023^^;^ ^Yuan & Jing, 2024^^).^

#### 2.3.4 Entropy analysis (EA)

The entropy method (EA) is an analytical technique used primarily to measure diversity or disorder within a system and is particularly useful in decision-making processes where multiple criteria are involved. Using the entropy method, five types of teas were weighted to calculate the comprehensive score S values (L. B. Zhou et al., 2022).

(1) Standardized treatment,$y{'}_{\mathrm{ij}}=\frac{x_{\mathrm{ij}}-x_{j\min}}{x_{j\max}-x_{j\min}}$, where$y{'}_{\mathrm{ij}}$(*i* = 1,2…, n; j = 1,2…, m) is the j index value of sample i after dimensionless treatment, and the original data of the j index of sample i are the maximum value of the j index and the minimum values of the j index.
(2) Calculation of the proportion of sample i under indicator j$P_{\mathrm{ij}}\left(0\leq P_{\mathrm{ij}}\leq 1\right)P_{\mathrm{ij}}=\frac{y{'}_{\mathrm{ij}}}{\sum_{i=1}^{n}y{'}_{\mathrm{ij}}}$
(3) Information entropy value e and information utility value d, the information entropy value of item j is $e_{j}=-\frac{1}{\ln m}\sum_{i=1}^{n}P_{\mathrm{ij}}{\mathrm{lnP}}_{\mathrm{ij}}$ Information utility value$d_{j}=1-e_{j}$
(4) Weight of the evaluation indicators. The greater the information utility value is, the more important the indicators are, and the greater the importance of evaluation is. Finally, the weight of the jth index is$W_{j}=\frac{d_{j}}{\sum_{j=1}^{m}d_{j}}$
(5) Comprehensive evaluation$S=\sum_{j=1}^{m}\left(W_{j}P_{\mathrm{ij}}\right)$

#### 2.3.5 Factor cluster analysis (FCA)

Factor cluster analysis (FCA) is a statistical approach used to group variables according to their similarities or relationships. It is a mix of factor analysis and cluster analysis in which the variables are first reduced to a smaller number of factors via factor analysis and then clustered based on their similarities using cluster analysis The dimension of the original data is reduced according to factor analysis, and a few common factors are extracted from a large number of index variables. Under the principle of reducing information loss as much as possible, the information of the original variables is replaced by new common factor variables(Cuppen et al. Cuppen et al., 2018; Okrent et al., 2017).

## 3 Results and discussion

### 3.1 Determination of the combustion heat of tea

#### 3.1.1 ^The combustion heat of Duyun Maojian tea^

According to the calculations, ∆m_Duyun Maojian tea_Qv_Duyun Maojian tea_ = W_cal_∆T-Q_ignition wire_∆m_ignition wire-Qcapsule_∆m_capsule_, W_cal_ = 14,200.79 J/°C, ∆*T* = 0.345 °C, Q_ignition wire_ = 1400.8 J/g, Q_capsule_ = 43,284.83 J/g, ∆m_ignition wire_ = 0.0098 g, ∆m_capsule_ = 0.0989 g, and ∆m_Duyun Maojian tea_ = 0.1359 g. The average Qv_Duyun Maojian tea_ content is 4449.4115 J/g.

#### 3.1.2 Determination of the combustion heat of other tea leaves

**Fig. 1:**
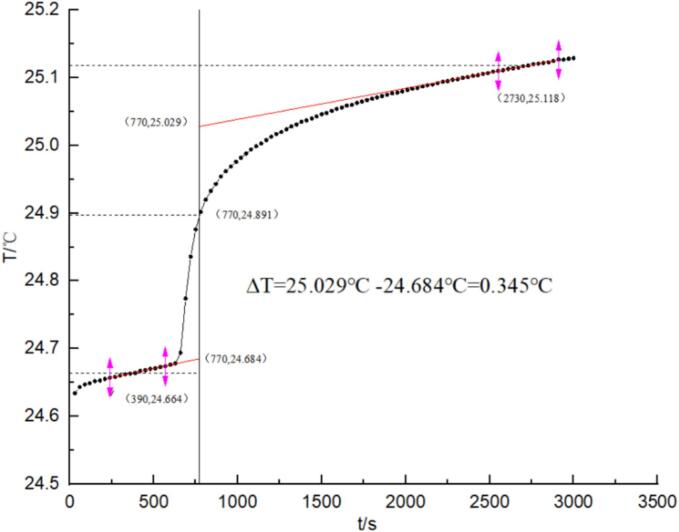
Reynolds temperature ∆T curve of Duyun Maojian tea.

Similarly, the combustion heats of Liping fragrant tea, Shuicheng spring tea, Huaxi jasmine tea, and Zunyi black tea were determined, and the test was repeated three times. The combustion heats of the five tea varieties are presented in Table 1. Table 1 shows the combustion heat of the five tea samples, which decreased in the following order: Liping fragrant tea > Shuicheng spring tea > Duyun Maojian tea > Zunyi black tea > Huaxi jasmine tea. The combustion heat of the five teas ranged from 2610.18 to 5849.31 J/g. The highest combustion heat was exhibited by the Liping fragrant tea, with a value of 5849.308 J/g. In comparison, Shuicheng spring tea, Duyun Maojian tea, Zunyi black tea and Huaxi jasmine tea displayed combustion heats of 4932.178, 4475.976, 3172.202, and 2610.184 J/g, respectively. The energy released by these processes was insignificant. Accordingly, when assessing the quality of tea based on combustion heat, high-quality tea typically displays greater combustion heat and less residue, which is indicative of its inherent quality. (See Fig. 1.)

**Table 1:** Combustion heat of five kinds of tea (*n* = 3).

Sample	Q_Vaverage_/(J/g)	RSD/%
Duyun Maojian tea	4475.976 ± 220.952	4.9364
Liping fragrant tea	5849.308 ± 279.018	4.7701
Shuicheng spring tea	4932.178 ± 245.553	4.9786
Huaxi jasmine tea	2610.184 ± 117.552	4.5036
Zunyi black tea	3172.202 ± 157.011	4.9496

### 3.2 Thermogravimetric analysis

#### 3.2.1 Duyun Maojian tea

The thermogravimetric data for the Duyun Maojian tea are presented in Fig. 2a and Table 2a. The pyrolysis process of the Duyun Maojian samples underwent three main stages.The Duyun Maojian tea sample began to decompose at 39.9 °C, at this stage there may be a small loss of sample quality due to the release of moisture from the sample.After the first stage of decomposition, the loss rate was 5.13 %. Upon reaching 168.4 °C, the sample entered the second stage of decomposition. Upon reaching a temperature of 401.5 °C, the reason for the sharp decrease in mass at this stage may be the breakdown of components such as sugars, amino acids, and fats and oils, with a loss rate of 41.33 %.The third stage of decomposition may be the loss of crude fiber and other components, with a loss rate of 11.58 %.The sample continued to decompose, with a final remaining mass of 41.54 %. The DTG curve for Duyun Maojian tea exhibited two peaks with inflection points at 95.2 °C and 336.0 °C. The DTA curve of Duyun Maojian tea displays a pronounced exothermic peak with a maximum temperature of 116.5 °C. The temperature range encompassing this peak was 71.1 °C to 183.5 °C, with a peak area of 138.4 J/g. Additionally, an endothermic peak was observed, with a maximum temperature of 353.5 °C and a temperature range of 320.2 °C to 404.3 °C, exhibiting a peak area of 26.82 J/g.

**Fig. 2a:**
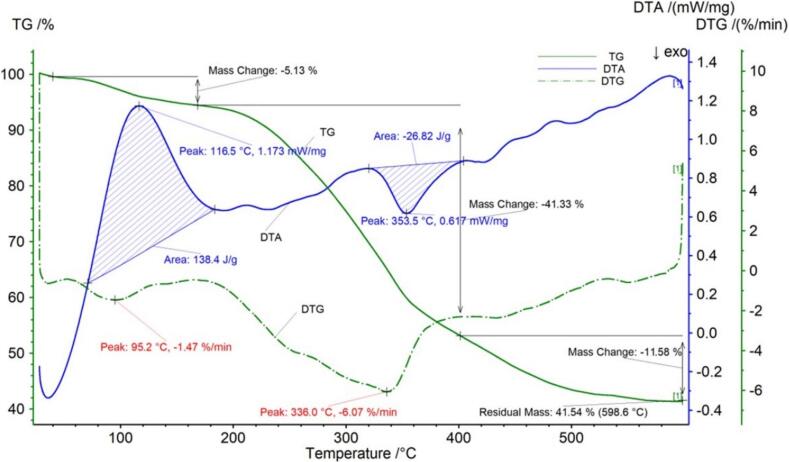
Thermogravimetric (TG) curve, derivative thermogravimetric (DTG) curve and differential thermal analysis (DTA) curve of Duyun Maojian tea.

**Table 2a:** Thermogravimetric parameters of Duyun Maojian tea.

Curve	Project	Temperature range/°C	Percentage weight/%	Peak area/J/g	The fastest weight loss temperature/°C
TG，DTG	Peak1	39.9–168.4	5.13	–	95.2
	Peak2	168.4–401.5	41.33	–	336.0
DTA	Peak1	71.1–183.5	–	138.40	95.2
	Peak2	320.2–404.3	–	26.82	336.0

#### 3.2.2 Liping fragrant tea

The thermogravimetric data for the Liping fragrant tea are shown in Fig. 2b and Table 2b. Fig. 2b shows that the Liping fragrant tea sample began to decompose at 43.3 °C, and there may be a small loss of sample quality due to the release of moisture from the sample. The loss rate after the first decomposition stage was 5.30 %. When the temperature reached 169.8 °C, the tea entered the second stage of decomposition. When the temperature reached 401.5 °C, the reason for the sharp decrease in mass at this stage may be the breakdown of components such as sugars, amino acids, and fats and oils, with a loss rate of 39.31 %. The third stage of decomposition may be the loss of crude fiber and other components, with a loss rate of 11.45 %.The sample continued to decompose and the final mass remained at 43.29 %. The DTG curve of the Liping fragrant tea showed two peaks, with inflection points at 100.2 °C and 334.9 °C. The DTA curve of the Liping fragrant tea showed a large exothermic peak with a peak value of 114.7 °C, a temperature range of 66.8 °C- 170.5 °C, and a peak area of 129.00 J/g. There was an endothermic peak, with a peak value of 352.5 °C, a temperature range of 324.6 °C–402.5 °C, and a peak area of 38.58 J/g.

**Fig. 2b:**
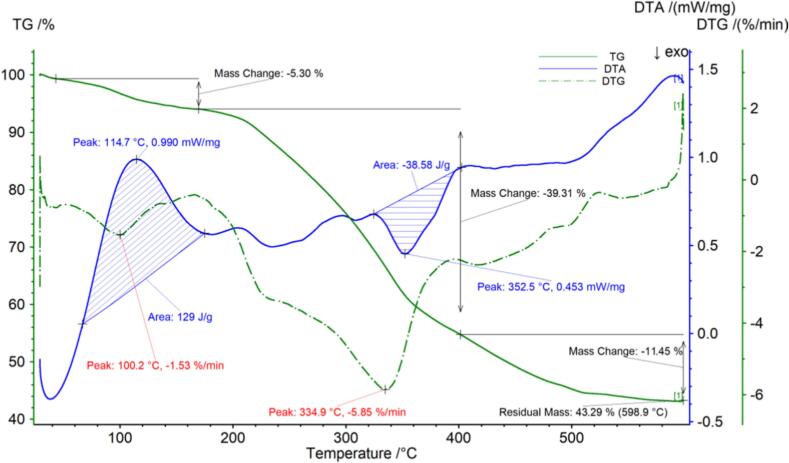
Thermogravimetric (TG) curve, derivative thermogravimetric (DTG) curve and differential thermal analysis (DTA) curve of Liping fragrant tea.

**Table 2b:** Thermogravimetric parameters of the Liping fragrant tea.

Curve	Project	Temperature range/°C	Percentage weight /%	Peak area/J/g	The fastest weight loss temperature/°C
TG，DTG	Peak1	43.3–169.8	5.30	–	100.2
	Peak2	169.8–401.5	39.31	–	334.9
DTA	Peak1	66.8–175.0	–	129.00	114.7
	Peak2	324.6–402.5	–	38.58	352.5

#### 3.2.3 Shuicheng spring tea

The thermogravimetric data for Shuicheng spring tea are shown in Fig. 2c and Table 2c. Fig. 2c shows that the temperature at which the Shuicheng spring tea sample began to decompose was 44.6 °C, at this stage there may be a small loss of sample quality due to the release of moisture from the sample. After the first decomposition stage, the loss rate was 5.01 %. When the temperature reached 145.3 °C, the sample entered the second stage of decomposition. When the temperature reached 401.9 °C, the reason for the sharp decrease in mass at this stage may be the breakdown of components such as sugars, amino acids, and fats and oils, with a loss rate of 47.63 %. The third stage of decomposition may be the loss of crude fiber and other components, with a loss rate of 12.55 %. The sample continued to decompose, and the final remaining mass was 34.23 %. The DTG curve of Shuicheng spring tea showed two peaks, with inflection points at 103.0 °C and 335.5 °C. The DTA curve of Shuicheng spring tea has a large exothermic peak, with a peak value of 110.1 °C, a temperature range of 44.6 °C - 19.1 °C, and a peak area of 239.2 J/g.

**Fig. 2c:**
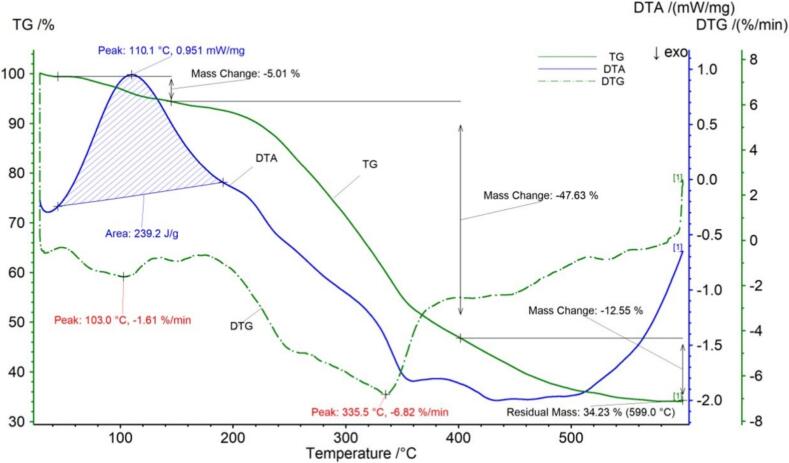
Thermogravimetric (TG) curve, derivative thermogravimetric (DTG) curve and differential thermal analysis (DTA) curve of Shuicheng spring tea.

**Table 2c:** Thermogravimetric parameters of Shuicheng spring tea.

Curve	Project	Temperature range/°C	Percentage weight /%	Peak area/J/g	The fastest weight loss temperature/°C
TG，DTG	Peak1	44.6–145.3	5.01	–	103.0
	Peak2	145.3–401.9	47.63	–	335.5
DTA	Peak1	44.6–191.1	–	239.2	110.1
	Peak2	–	–	–	–

#### 3.2.4 Huaxi jasmine tea

The thermogravimetric data for Huaxi jasmine tea are shown in Fig. 2d and Table 2d. Fig. 2d shows that the temperature at which the Huaxi jasmine tea sample began to decompose was 42.5 °C, at this stage there may be a small loss of sample quality due to the release of moisture from the sample. After the first decomposition stage, the loss rate was 6.17 %. When the temperature reached 172.5 °C, the tea entered the second stage of decomposition. When the temperature reached 407.1 °C, the reason for the sharp decrease in mass at this stage may be the breakdown of components such as sugars, amino acids, fats, and oils, with a loss rate of 45.41 %. The third stage of decomposition may be the loss of crude fiber and other components, with a loss rate of 12.77 %. The sample continued to decompose, and the final remaining mass was 35.25 %. The DTG curve of Huaxi jasmine tea showed two peaks, with inflection points at 97.6 °C and 335.3 °C. The DTA curve of Huaxi jasmine tea exhibited a large exothermic peak; the peak value was 110.2 °C, the temperature range was 37.8 °C–190.1 °C, and the peak area was 251.2 J/g. There was also an endothermic peak with a peak value of 358.8 °C, a temperature range of 329.0–394.8 °C, and a peak area of 25.06 °C.

**Fig. 2d:**
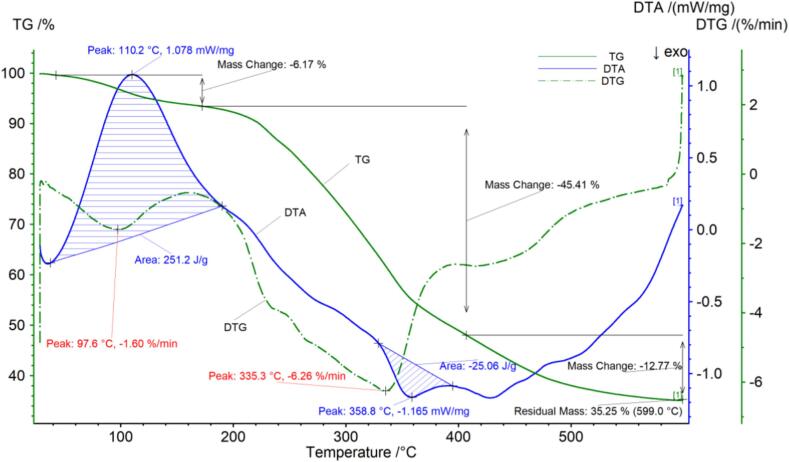
Thermogravimetric (TG) curve, derivative thermogravimetric (DTG) curve and differential thermal analysis (DTA) curve of Huaxi jasmine tea.

**Table 2d:** Thermogravimetric parameters of Huaxi jasmine tea.

Curve	Project	Temperature range/^°C^	Percentage weight /%	Peak area/J/g	The fastest weight loss temperature/^°C^
TG，DTG	Peak1	42.5–172.5	6.17	–	97.6
	Peak2	172.5–407.1	45.41	–	335.3
DTA	Peak1	37.8–190.1	–	251.2	110.2
	Peak2	329.0–394.8	–	25.06	358.8

#### 3.2.5 Zunyi black tea

The thermogravimetric data for Zunyi black tea are presented in Fig. 2e and Table 2e. Fig. 2e shows that the temperature at which decomposition of the Zunyi black tea sample commenced was 58.3 °C, at this stage there may be a small loss of sample quality due to the release of moisture from the sample. The loss rate was 2.27 % following the initial phase of decomposition. At 144.3 °C, the process entered its second stage of decomposition. At 447.1 °C, the reason for the sharp decrease in mass at this stage may be the breakdown of components such as sugars, amino acids, and fats and oils, with a loss rate of 42.35 %. The third stage of decomposition may be the loss of crude fiber and other components, with a loss rate of 7.37 %.The sample continued to decompose with a final remaining mass of 46.33 %. The DTG curve of Zunyi black tea exhibited two peaks, with inflection points at 118.7 °C and 339.1 °C. The DTA curve of Zunyi black tea exhibited an exothermic peak with a peak value of 110.7 °C, a temperature range of 69.2 °C to 182.5 °C, and a peak area of 211.1 J/g.

**Fig. 2e:**
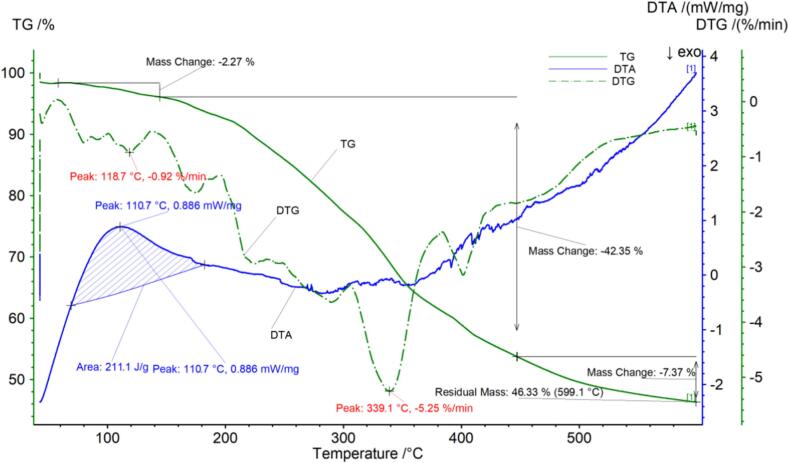
Thermogravimetric (TG) curve, derivative thermogravimetric (DTG) curve and differential thermal analysis (DTA) curve of Zunyi black tea.

**Table 2e:** Thermogravimetric parameters of Zunyi black tea.

Curve	Project	Temperature range/	Percentage weight /%	Peak area/J/g	The fastest weight loss temperature/^°C^
TG，DTG	Peak1	58.3–144.3	2.27	–	118.7
	Peak2	144.3–447.1	42.35	–	339.1
DTA	Peak1	69.2–182.5	–	211.1	110.7
	Peak2	–	–	–	–

#### 3.2.6 Combustion stability analysis of the five kinds of tea samples

According to the combustion parameter data of Duyun Maojian tea, Liping fragrant tea, Shuicheng spring tea, Huaxi jasmine tea, and Zunyi black tea, a combustion stability multi-index evaluation system was constructed. EXCEL calculations were performed to assess the combustion stability of the various teas. The results indicated that Duyun Maojian, Liping fragrant tea, Shuicheng spring tea, Huaxi jasmine tea, and Zunyi black teas had combustion stabilities of 0.8252, 0.8759, 0.8399, 0.8956, and 0.7902, respectively. The order of the combustion stability parameter data for the five types of tea is as follows: Huaxi jasmine tea>Liping fragrant tea>Shuicheng spring tea>Duyun Maojian tea >Zunyi black tea. These values reflect the thermal behavior of each tea type under the conditions tested.

### 3.3 Results analysis of fat, catechins, crude fiber, etc

The results of the analysis of the fat, catechin, crude fiber, etc., contents of the five tea leaves are presented in Table 3. The fat content ranged from 0.20 % to 0.65 %, with an average of 0.37 %. The order of magnitude was as follows: Liping fragrant tea > Duyun Maojian tea > Zunyi black tea > Huaxi jasmine tea > Shuicheng spring tea. The ash content ranged from 4.75 % to 5.58 %, with an average value of 5.21 %. The order of magnitude was as follows: Duyun Maojian tea > Huaxi jasmine tea > Shuicheng spring tea > Liping fragrant tea > Zunyi black tea. The crude fiber content ranged from 11.07 % to 13.37 %, with an average value of 12.42 %. The order of magnitude was as follows: Liping fragrant tea > Zunyi black tea > Duyun Maojian tea > Huaxi jasmine tea > Shuicheng spring tea. Evaluating indicators such as the fat content, crude fiber content, catechin content, theanine and caffeine contents, and ash content of tea can help provide a comprehensive understanding of the nutritional composition and quality characteristics of tea (Alasalvar et al., 2013; Oanh et al., 2023; Serpen et al., 2012). These indicators reflect the freshness, processing quality, and purity of tea leaves, helping consumers select high-quality tea leaves. These indicators are also important for identifying tea quality and can help producers and regulatory authorities control and monitor tea quality. Therefore, the evaluation of these indicators can play an important role in the tea industry (L. Zhou et al., 2023; M. Zhou et al., 2024).

**Table 3:** Results of the determination of the fat, ash and crude fiber contents of 5 kinds of tea leaves (*n* = 5).

Sample	Fat/%	Ash/%	Crude fiber/%	Catechins/%	Theanine/(g/kg)	Caffeine/(mg/g)
Duyun Maojian tea	0.3804 ± 0.0132	5.5766 ± 0.2708	12.9194 ± 0.2856	12.5967 ± 0.0351	10.2923 ± 0.0704	19.7287 ± 0.1087
Liping fragrant tea	0.6401 ± 0.025	4.9763 ± 0.2155	13.3696 ± 0.5336	11.3103 ± 0.0261	6.7297 ± 0.1078	19.4353 ± 0.1895
Shuicheng spring tea	0.208 ± 0.0042	5.2204 ± 0.2357	11.0798 ± 0.5122	10.5924 ± 0.0275	4.8187 ± 0.0542	13.466 ± 0.1735
Huaxi jasmine tea	0.2524 ± 0.0073	5.5001 ± 0.272	11.731 ± 0.4484	0.746 ± 0.0111	1.8278 ± 0.0135	96.377 ± 0.0525
Zunyi black tea	0.3692 ± 0.0117	4.7557 ± 0.0238	13.0064 ± 0.4164	16.1053 ± 0.1899	7.0592 ± 0.0845	15.4883 ± 0.1488

### 3.4 Determination of trace element contents

#### 3.4.1 Results for the trace elements

A method using inductively coupled plasma–optical emission spectrometry (ICP–OES) (Pohl et al., 2018; Pohl et al., 2022) based on microwave digestion was developed to analyze 17 trace elements in five types of tea from Guizhou, China: Duyun Maojian tea, Liping fragrant tea, Shuicheng spring tea, Huaxi jasmine tea and Zunyi black tea. The study provides the optimum wavelengths, correlation coefficients (R) and detection limits of the instruments (Cantlay et al., 2020; Chojnacka et al., 2018) for the elements, as shown in Table s4a. The evaluation results were deemed reasonable, thereby highlighting the significance of this approach. By providing valuable details regarding the trace element compositions of various teas, this study advances our understanding of their nutritional and elemental characteristics. The results of the trace element analysis are presented in Table s4b. As shown in Table s4b, none of the five types of tea contained Hg or Sc.

#### 3.4.2 Gray principal component analysis (GPCA) of the trace element determination results

Table s4c shows the eigenvalues of the gray correlation matrix and the variance of the contribution rate obtained. The cumulative contribution ratio of the first three gray principal components was 94.170 %, which was greater than 85 %. These findings indicate that these components are highly representative of the variation in the 15 trace elements present in the five types of tea. The characteristic roots (λ > 1.000) were identified as λ_1_ = 7.152, λ_2_ = 4.043, and λ_3_ = 2.931, all of which exceeded 1.000. Consequently, the first three gray principal components were selected to represent 94.170 % of the variation in trace elements across the five types of tea.

**Fig. 3:**
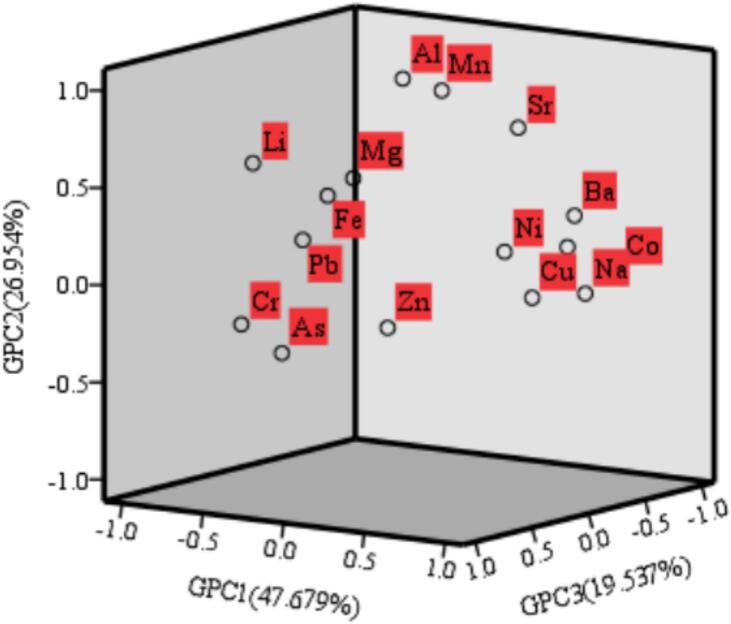
Gray principal component analysis of the 15 trace elements in the five different teas.

An overview of the original variables is provided by the initial solution obtained via gray principal component analysis (GPCA), as shown in Fig. 3. Moreover, it shows the result of orthogonally rotating the gray correlation coefficient matrix with the maximum variance. Because it shows the underlying structure of the data, this visualization is crucial for understanding the correlations and patterns between the original variables and gray principle components (M. Y. Zhao, Gou, et al., 2023).

The first gray principal component (Table s4d) predominantly comprises data pertaining to Ba, Co, Cu, Na and Ni. The Ba content ranged from 9.05 to 15.65 μg/g, with an average content of 11.31 μg/g, in the following order: Shuicheng spring tea > Huaxi jasmine tea > Zunyi black tea > Duyun Maojian tea > Liping fragrant tea. The Co content ranged from 0.25 to 1.17 μg/g, with an average content of 0.49 μg/g, and the order of the contents was Shuicheng spring tea > Huaxi jasmine tea > Liping fragrant tea > Zunyi black tea > Duyun Maojian tea. The Cu content ranged from 33.23 to 58.78 μg/g, with an average content of 41.10 μg/g, in the following order: Shuicheng spring tea > Duyun Maojian tea > Zunyi black tea > Liping fragrant tea > Huaxi jasmine tea. The Na content ranged from 254.43 to 358.71 μg/g, with an average content of 307.83 μg/g, in the following order: Shuicheng spring tea > Zunyi black tea > Huaxi jasmine tea > Duyun Maojian tea > Liping fragrant tea. The Ni content ranged from 7.04 to 15.86 μg/g, with an average content of 10.59 μg/g, and the Ni content decreased in the following order: Shuicheng spring tea > Duyun Maojian tea > Huaxi jasmine tea > Liping fragrant tea > Zunyi black tea.

The second gray principal component is primarily composed of data pertaining to the concentrations of Al, Fe, Li, Mg, Mn and Sr. The content of Al ranged from 185.42 to 693.36 μg/g, with an average content of 382.18 μg/g, and the content decreased in the following order: Huaxi jasmine tea > Shuicheng spring tea > Liping fragrant tea > Duyun Maojian tea > Zunyi black tea. The Fe content ranged from 167.89 to 248.15 μg/g, with an average content of 213.51 μg/g, in the following order: Huaxi jasmine tea > Zunyi black tea > Liping fragrant tea > Duyun Maojian tea > Shuicheng spring tea. The Li content ranged from 0.07 to 0.15 μg/g, with an average content of 0.13 μg/g, and the content decreased in the following order: Huaxi jasmine tea > Duyun Maojian tea > Liping fragrant tea > Zunyi black tea > Shuicheng spring tea. The teas containing Mg were Huaxi jasmine tea, Liping fragrant tea and Zunyi black tea. The Mn content ranged from 398.15 to 918.52 μg/g, with an average value of 641.76 μg/g, and the content decreased in the following order: Huaxi jasmine tea > Shuicheng spring tea > Liping fragrant tea > Duyun Maojian tea > Zunyi black tea. The Sr content ranged from 3.07 to 5.64 μg/g, with an average content of 4.13 μg/g, and the content decreased in the following order: Shuicheng spring tea > Huaxi jasmine tea > Duyun Maojian tea > Zunyi black tea > Liping fragrant tea.

The third gray principal component mainly contained information on As, Cr, Pb, and Zn. The teas containing As were Liping fragrant tea and Duyun Maojian tea. The Cr content ranged from 1.02 to 2.12 μg/g, with an average content of 1.56 μg/g, in the order of Liping fragrant tea > Duyun Maojian tea > Huaxi jasmine tea > Zunyi black tea > Shuicheng spring tea. The Pb content ranged from 0.99 to 2.50 μg/g, with an average content of 1.67 μg/g, and the order of Pb content was Duyun Maojian tea > Huaxi jasmine tea > Shuicheng spring tea > Liping fragrant tea > Zunyi black tea. The Zn content ranged from 157.01 to 319.99 μg/g, with an average content of 243.89 μg/g, and the content decreased in the following order: Duyun Maojian tea > Shuicheng spring tea > Liping fragrant tea > Huaxi jasmine tea > Zunyi black tea.

The gray principal component was represented by the following linear regression equation for each variable: f_1_ = −0.093 * Al-0.156 * As +0.911 * Ba+0.936 * Co + 0.699 * Cr + 0.958 * Cu-0.802 * Fe-0.824 * Li-0.728 * Mg + 0.208 * Mn + 0.802 * Na + 0.874 * Ni - 0.040 * Pb + 0.585 * Sr + 0.467 * Zn; f_2_ = 0.993 * Al-0.284 * As +0.377* Ba+0.233 * Co + 0.250 * Cr + 0.024 * Cu + 0.269 * Fe + 0.525 * Li + 0.349 * Mg + 0.974 * Mn - 0.070 * Na + 0.269 * Ni + 0.307 * Pb + 0.801 * Sr - 0.097 * Zn; f_3_ = − 0.077 * Al + 0.903 * As - 0.162* Ba - 0.063 * Co + 0.486 * Cr + 0.280 * Cu - 0.424 * Fe + 205 * Li - 0.545 * Mg + 0.009 * Mn - 0.413 * Na + 0.405 * Ni +0.884 * Pb - 0.129 * Sr + 0.858 * Zn;Composite gray principal component scores: f = 0.47679* f_1_ + 0.26954* f_2_ + 0.19537* f_3_.

The gray principal component scores and composite gray principal component scores (Table s4e) of the 15 trace elements, including Al, As, Ba, Co, Cr, Cu, Fe, Li, Mg, Mn, Na, Ni, Pb, Sr, and Zn, revealed that the trace element concentrations of the five teas were as follows: Shuicheng spring tea > Huaxi jasmine tea > Duyun Maojian tea > Liping fragrant tea > Zunyi black tea. In terms of trace element contents, Shuicheng spring tea had the highest quality, followed by Huaxi jasmine tea.

### 3.5 Determination of amino acids in tea leaves

#### 3.5.1 Amino acid determination results

**Fig. 4:**
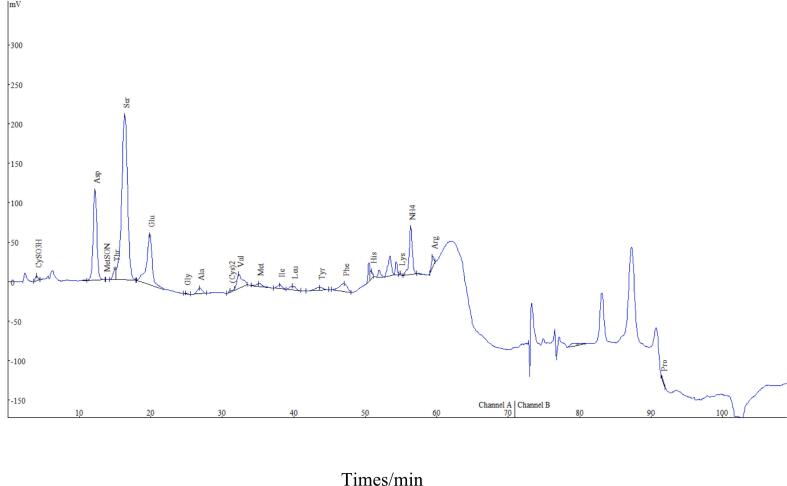
The peak spectrum of the amino acid content of Duyun Maojian tea.

In Guizhou, China, the development of an amino acid analyzer method based on ninhydrin activation represents a significant advance in the determination of 20 amino acids found in five types of tea: Duyun Maojian tea, Liping fragrant tea, Shuicheng spring tea, Huaxi jasmine tea, and Zunyi black tea. This approach has significant potential for providing thorough insights into the amino acid content of these teas, leading to a more complete understanding of their nutritional profiles and potential health advantages. Fig. 4 shows the peak spectrum of the amino acid content (El-Sohaimy et al., 2022; Malik & Riar, 2022; Venmarath et al., 2024) of Duyun Maojian tea, and Table s5a presents the amino acid determination results for the five teas. The ratio of the human essential amino acid content to the total amino acid content ranged from 12.52 % to 33.38 %, whereas the ratio of the human essential amino acid content to the nonessential amino acid content (Cho et al., 2024; Effiong et al., 2023; X. Zhao, Zhang, et al., 2023) ranged from 14.13 % to 50.11 %.

#### 3.5.2 Gray factor analysis (GFA)

After performing gray factor analysis, the cumulative contribution (Table s5b) of the first four gray factors reached 100.00 % > 85 %; therefore, the first four gray factors were selected, which represented 100.00 % of the 19 amino acids(Ayuso et al., 2024; Bishani et al., 2024; P. Zhang, Xu, & Zhou, 2024), including CySO_3_H, Asp, Thr, Ser, Glu, Gly, Ala, (Cys)_2_, Val, Met, Ile, Leu, Tyr, Phe, His, Lys, NH_4_, Arg and Pro, in five teas in Guizhou, China: Duyun Maojian tea, Liping fragrant tea, Shuicheng spring tea, Huaxi jasmine tea, and Zunyi black tea.

The first gray factor loading matrix (Table s5c) contains information on Asp, Thr, Ala, Val, Met, Ile, Leu, Tyr, Phe, His, Lys, Arg and Pro. The Asp content ranged from 136.06 to 3439.06 μg/g, with a mean value of 2153.31 μg/g, and the order of content was Shuicheng spring tea > Liping fragrant tea > Duyun Maojian tea > Huaxi jasmine tea > Zunyi black tea. The content of Thr ranged from 8.39 to 460.80 μg/g, with a mean value of 288.85 μg/g, and the content decreased in the following order: Shuicheng spring tea > Liping fragrant tea > Huaxi jasmine tea > Duyun Maojian tea > Zunyi black tea. The Ala content ranged from 23.34 to 295.72 μg/g, with a mean value of 186.36 μg/g, and the content decreased in the following order: Shuicheng spring tea > Huaxi jasmine tea > Duyun Maojian tea > Liping fragrant tea > Zunyi black tea. The val content ranged from 31.93 to 995.65 μg/g, with a mean value of 416.93 μg/g, and the content decreased in the following order: Shuicheng spring tea > Duyun Maojian tea > Liping fragrant tea > Huaxi jasmine tea > Zunyi black tea. The Met content ranged from 190.22 to 612.70 μg/g, with a mean value of 288.22 μg/g, and the order of content was Shuicheng spring tea > Zunyi black tea > Huaxi jasmine tea > Liping fragrant tea > Duyun Maojian tea. The Ile content ranged from 24.19 to 391.35 μg/g, with a mean value of 203.32 μg/g, and the content decreased in the order Shuicheng spring tea > Liping fragrant tea > Huaxi jasmine tea > Duyun Maojian tea > Zunyi black tea. The Leu content ranged from 127.36 to 1338.22 μg/g, with a mean value of 437.26 μg/g, and the content decreased in the following order: Shuicheng spring tea > Liping fragrant tea > Huaxi jasmine tea > Duyun Maojian tea > Zunyi black tea. The Tyr content ranged from 147.44 to 472.16 μg/g, with a mean value of 312.29 μg/g, and the content decreased in the order Shuicheng spring tea > Liping fragrant tea > Huaxi jasmine tea > Duyun Maojian tea > Zunyi black tea. The Phe content ranged from 482.98 to 936.76 μg/g, with a mean value of 642.25 μg/g, and the content decreased in the order Shuicheng spring tea > Liping fragrant tea > Duyun Maojian tea > Zunyi black tea > Huaxi jasmine tea.His content ranged from 21.71 to 165.14 μg/g, and the order of content was Shuicheng spring tea > Duyun Maojian tea > Huaxi jasmine tea > Zunyi black tea, while it was not detected in the Liping fragrant tea. The Lys content ranged from 20.49 to 676.38 μg/g, with an average value of 203.35 μg/g, and the contents decreased in the following order: Shuicheng spring tea > Huaxi jasmine tea > Zunyi black tea > Duyun Maojian tea > Liping fragrant tea. The Arg content ranged from 180.33 to 1367.73 μg/g, with a mean value of 665.57 μg/g, and the content decreased in the order Shuicheng spring tea > Zunyi black tea > Huaxi jasmine tea > Duyun Maojian tea > Liping fragrant tea. The Pro content ranged from 12.53 to 255.77 μg/g, with an average value of 70.92 μg/g, and the content decreased in the following order: Shuicheng spring tea > Liping fragrant tea > Duyun Maojian tea > Huaxi jasmine tea > Zunyi black tea.

The second gray factor loading matrix contained information on CySO_3_H, Ser, Gly, and NH_4_. The CySO_3_H content ranged from 93.38 to 186.49 μg/g, with an average value of 127.47 μg/g, and the content decreased in the order Shuicheng spring tea > Huaxi jasmine tea > Liping fragrant tea > Duyun Maojian tea > Zunyi black tea. The Ser content ranged from 385.76 to 13,757.67 μg/g, with a mean value of 6682.09 μg/g, and the order of content was Shuicheng spring tea > Huaxi jasmine tea > Liping fragrant tea > Duyun Maojian tea > Zunyi black tea. The Gly content ranged from 4.23 to 13.11 μg/g, and the content decreased in the following order: Huaxi jasmine tea > Liping fragrant tea > Duyun Maojian tea > Zunyi black tea, while it was not detectable in Shuicheng spring tea. The NH_4_ content ranged from 58.49 to 138.72 μg/g, with a mean value of 101.89 μg/g, and the content decreased in the following order: Huaxi jasmine tea > Shuicheng spring tea > Duyun Maojian tea > Zunyi black tea > Liping fragrant tea.

The third gray factor loading matrix mainly contained information on Glu. The Glu content ranged from 54.17 to 2879.58 μg/g, with a mean value of 2041.01 μg/g, and the content decreased in the following order: Liping fragrant tea > Shuicheng spring tea > Huaxi jasmine tea > Duyun Maojian tea > Zunyi black tea.

The fourth gray factor loading matrix mainly contained information on (Cys)_2_. The content of (Cys)_2_ ranged from 12.69 to 91.58 μg/g, with a mean value of 49.16 μg/g, and the order of content was Duyun Maojian tea > Shuicheng spring tea > Liping fragrant tea > Huaxi jasmine tea > Zunyi black tea.

The weighted least-squares approach was used to determine the gray factor scores (F_1_-F_4_) and composite gray factor scores (F), with the weights being the eigenroots of the gray factors. The formula used was F = 0.605 F_1_ + 0.2319 F_2_+ 0.0879 F_3_+ 0.0752 F_4_. The gray factor scores and composite gray factor scores (Table s5d) of the 19 amino acids, including CySO_3_H, Asp, Thr, Ser, Glu, Gly, Ala, (Cys)_2_, Val, Met, Ile, Leu, Tyr, Phe, His, Lys, NH_4_, Arg and Pro, revealed that the amino acid content order of the five teas was as follows: Shuicheng spring tea > Huaxi jasmine tea > Duyun Maojian tea > Liping fragrant tea > Zunyi black tea. The Shuicheng spring tea had the highest quality, followed by the Huaxi jasmine tea.

### 3.6 Multi-index analysis of five kinds of tea

#### 3.6.1 Entropy analysis (EA)

According to the combustion heat, combustion stability, fat, ash, crude fiber content, 15 trace element, content of catechins, theanine and caffeine, and 19 amino acid data for five types of tea, namely, Duyun Maojian tea, Liping fragrant tea, Shuicheng spring tea, Huaxi jasmine tea, and Zunyi black tea in Guizhou, China, a multi-index evaluation system was constructed via the entropy method (L. B. Zhou et al., 2022).

The S values of Duyun Maojian tea, Liping fragrant tea, Shuicheng spring tea, Huaxi jasmine tea, and Zunyi black tea in Guizhou, China, are 0.3791, 0.3102, 0.7041, 0.4242, and 0.1477, respectively. The nutritional value of the tea samples was assessed using a variety of indicators, including the heat of combustion, combustion stability, trace element content, amino acid content, fat content, ash content, and crude fiber content.

The entropy method was used to rank the nutritional values of the tea samples in the following order. A comprehensive evaluation revealed that the nutritional value of the tea samples decreased in the following order: Shuicheng spring tea > Huaxi jasmine tea > Duyun Maojian tea > Liping fragrant tea > Zunyi black tea. The Shuicheng spring tea is of the highest quality, and the Huaxi jasmine tea exhibits a similarly high standard.

#### 3.6.2 Factor cluster analysis (FCA)

Based on the heat of combustion data, thermogravimetric parameters, 15 trace element, fat, ash, crude fiber content, content of catechins, theanine and caffeine, and 19 amino acid contents of the five types of tea, namely, Duyun Maojian tea, Liping fragrant tea, Shuicheng spring tea, Huaxi jasmine tea, and Zunyi black tea, in Guizhou, China, factor cluster analysis (FCA) was applied to the five tea samples. Fig. 5a shows five tea samples, and factor cluster analysis (FCA) revealed that the samples could be classified into three categories.

**Fig. 5a:**
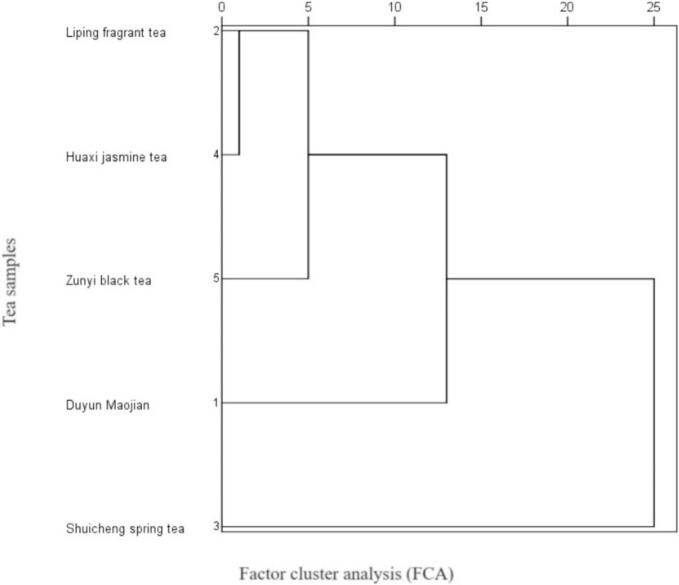
Factor cluster analysis (FCA) of 5 tea samples.

The Shuicheng spring tea fell into a single category, Liping fragrant tea, Huaxi jasmine tea and Zunyi black tea belonged to a category, as well as Duyun Mao Jian tea gathered into a category. The first category, consisting solely of Shuicheng spring tea, stands out as a unique entity. This isolation suggests that Shuicheng spring tea possesses distinctive chemical and nutritional properties that are distinct from those of the other samples. The unique profile of this tea warrants further investigation to identify the specific compounds or combinations of elements that contribute to its unique status. The second category, comprising Liping fragrant tea, Huaxi jasmine tea, and Zunyi black tea, demonstrated notable similarities in composition. Duyun Maojian tea, which forms the third category, presents distinct features that distinguish it from the other samples. This separation indicates that Duyun Maojian possesses a unique combination of chemical and nutritional attributes. This grouping revealed similarities among the five tea species on the Guizhou Plateau in China, laying the groundwork for future research in the field of food nutrition, particularly with respect to tea. The classification resulting from this factor cluster analysis serves as a valuable starting point for numerous research directions in food nutrition and related fields.

Factor cluster analysis (FCA) was conducted on 42 variables (Fig. 5b) based on a range of different indicators. The analysis revealed that the 42 variables could be classified into three groups. The variables Leu, Pro, Met, Co, Lys, Ba, CySO_3_H, Phe, Ser, Ile, Thr, Tyr, Asp, Glu, Gly, Li, caffeine, Ala, Al, Mn, Sr, Fe, Mg, and combustion stability were clustered into one category. The analysis primarily conveys information regarding amino acids and trace elements, which serve as fundamental building blocks of proteins and are highly nutritious. The variables Q_V_, fat, Cr, and crude fiber were grouped into a single category, primarily reflecting the energy properties of tea. Similarly, Arg, Na, catechins, Pb, Ash, NH_4_, (Cys)_2_, Zn, As, theanine, Val, Cu, His, and Ni were classified together, reflecting the heavy metal element data and the quality and safety of the tea. This classification provides insights into the nutritional composition, energy content, and safety aspects of the tea samples analyzed.

**Fig. 5b:**
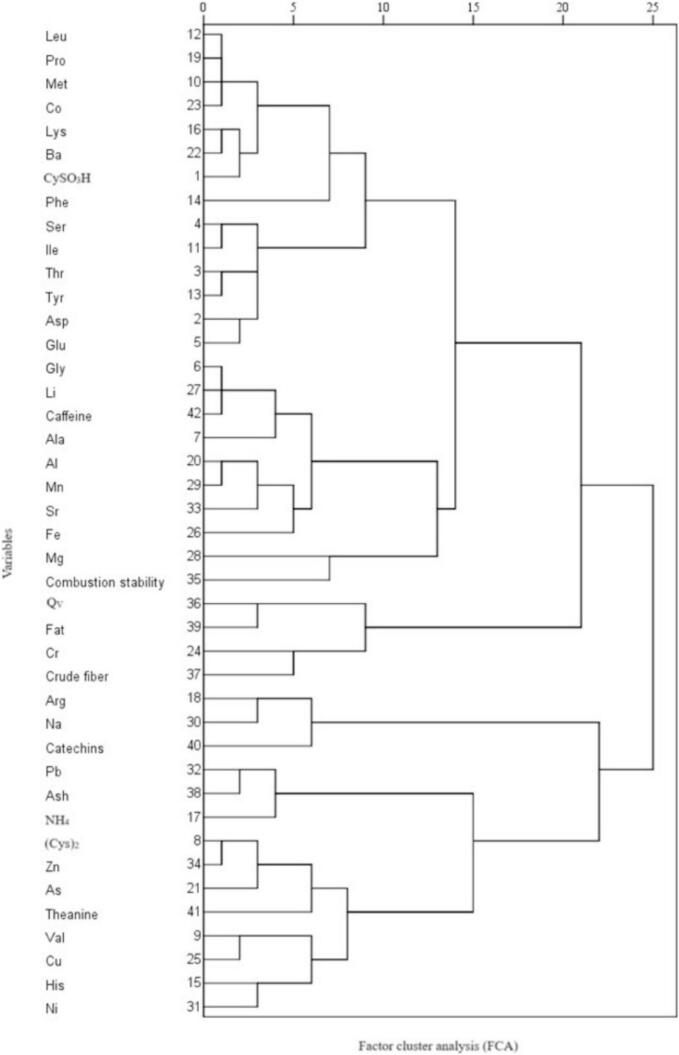
Factor cluster analysis (FCA) for 42 variables of 5 teas.

## 4 Conclusion

In this study, a comprehensive evaluation of 5 teas, namely, Duyun Maojian tea, Liping fragrant tea, Shuicheng spring tea, Huaxi jasmine tea, and Zunyi black tea, in Guizhou, China, in terms of combustion heat, combustibility, fat content, and amino acids (CySO_3_H, Asp, Thr, Ser, Glu, Gly, Ala, (Cys)_2_, Val, Met, Ile, Leu, Tyr, Phe, His, Lys, NH_4_, Arg, Pro), ash content, crude fiber content, content of catechins, theanine and caffeine, and trace elements (Al, As, Ba, Co, Cr, Cu, Fe, Li, Mg, Mn, Na, Ni, Pb, Sr, and Zn), was performed as follows: Shuicheng spring tea > Huaxi jasmine tea > Duyun Maojian tea >Liping fragrant tea > Zunyi black tea. The Shuicheng spring tea is the highest quality, and the Huaxi jasmine tea has a similarly high standard.

This study constructed comprehensive evaluation systems for five types of tea based on nutritional indicators, including multiple indicators of heat of combustion, combustibility (tea combustion stability), fat content, amino acid content, ash content, crude fiber content, catechin, theanine, caffeine, and trace element content, via gray pattern recognition (GPR), gray principal component analysis (GPCA), gray factor analysis (GFA), entropy analysis (EA), and factor cluster analysis (FCA). The new insight provided by this study is that it introduces a rigorous methodology for integrating various quality metrics, allowing for more detailed assessment and categorization of tea samples, and this integration could enhance strategies for improving tea quality. This study has significant theoretical and practical implications for the determination and comprehensive evaluation of multiple tea quality indicators. This study provides a scientific basis and research significance for nutritional, health, and classification studies on tea in Guizhou, China.

## CRediT authorship contribution statement

**Libing Zhou:** Writing – review & editing, Writing – original draft, Data curation. **Chunli Huang:** Writing – review & editing, Writing – original draft.

## Funding

This research was supported by the High-Level Talents Project of Guangxi Science & Technology Normal University (GXKS2020GKY006), Guangxi Science & Technology Normal University Key Laboratory of Speciality Food Evaluation and Application (GXKSKYPT2024011), and Guangxi Education Science “14th Five-Year Plan” 2024 Special Project “Construction and Application of Integrated Training System for Applied Talents of Food Quality and Safety in Ethnic Areas” (Project No. 2024ZJY1278).

## Declaration of competing interest

The authors declare that they have no known competing financial interests or personal relationships that could have appeared to influence the work reported in this paper.

## Data Availability

Data will be made available on request.

## Appendix A Supplementary data

Supplementary material Image 1
