# Supplementary material for: Stoichiometric analysis of 20 amino acids, thermogravimetric parameters and trace elements in five types of tea from Guizhou, China, based on entropy analysis (EA) and factor cluster analysis (FCA)
Source: Food Chem X. 2025 Apr 14;27:102457. doi: 10.1016/j.fochx.2025.102457 (PMC12131247; doi:10.1016/j.fochx.2025.102457)
Supplement: Supplementary file 1 — Supplementary material [file mmc1.docx]

Supplementary materials


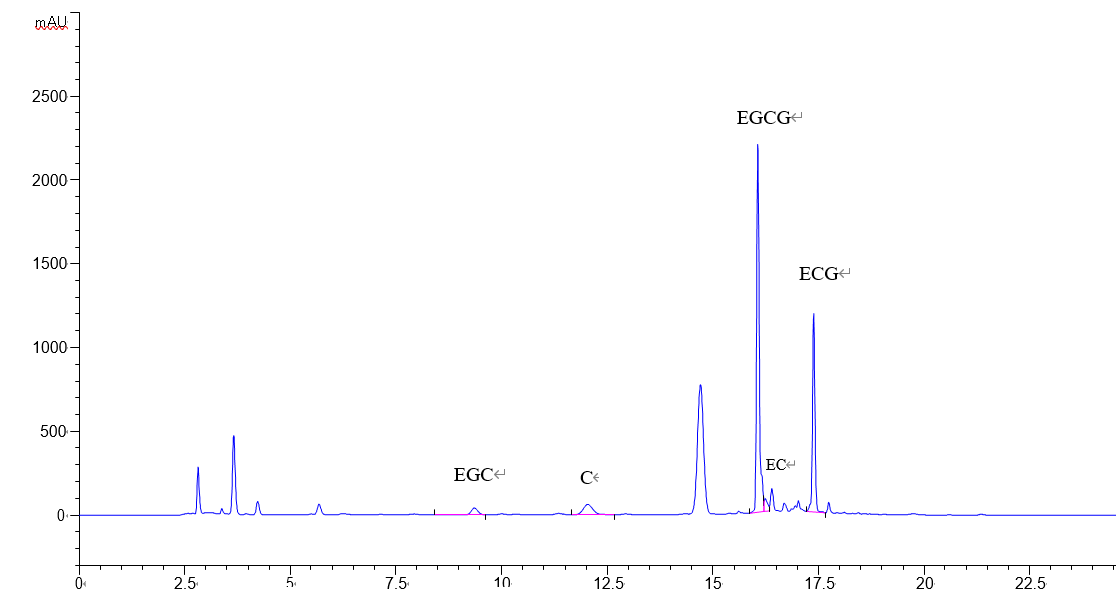


Times/min

Figure sa1 Catechins HPLC chromatogram of Duyun Maojian tea


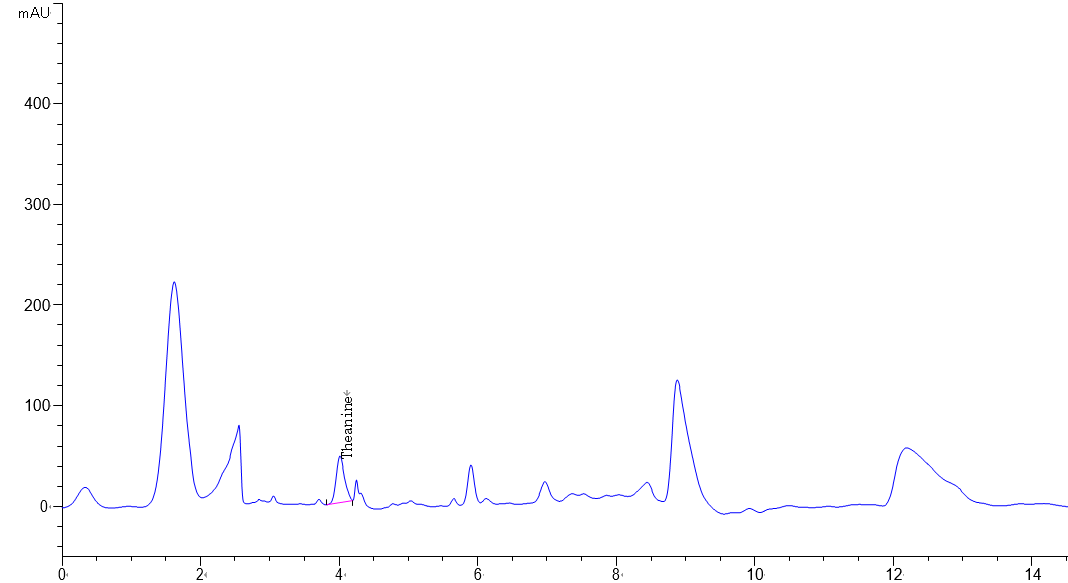


Times/min

Figure sa2 Theanine HPLC chromatogram of Duyun Maojian tea


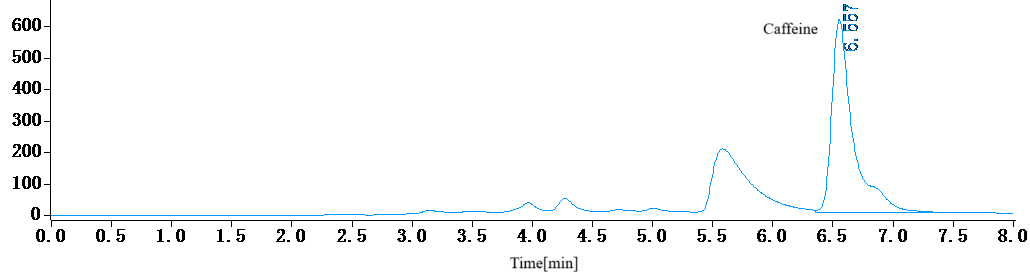


Figure sa3 Caffeine HPLC chromatogram of Duyun Maojian tea

Table Sa1 The thermal gravimetric parameter data of 5 teas

| Gravimetric parameters | Duyun Maojian tea | Liping fragrant tea | Shuicheng spring tea | Huaxi jasmine tea | Zunyi black tea |
| --- | --- | --- | --- | --- | --- |
| IndicatorX_1_/% | 5.13 | 5.3 | 5.01 | 6.17 | 2.27 |
| IndicatorX_2_/℃ | 95.2 | 100.2 | 103 | 97.6 | 118.7 |
| IndicatorX_3_/% | 41.33 | 39.31 | 47.63 | 45.41 | 42.35 |
| IndicatorX_4_/℃ | 336 | 334.9 | 335.5 | 335.3 | 339.1 |
| IndicatorX_5_/% | 11.58 | 11.45 | 12.55 | 12.77 | 7.37 |
| IndicatorX_6_/% | 41.54 | 43.29 | 34.23 | 35.25 | 46.33 |
| IndicatorX_7_/J/g | 138.4 | 129 | 239.2 | 251.2 | 211.1 |
| IndicatorX_8_/J/g | 26.82 | 38.58 | — | 25.06 | — |

Table s4a The optimum wavelengths, correlation coefficients (R) and detection limits of the instruments for the elements

| Element | Analyzing Wavelength/nm | Correlation coefficient | MDL/μg/ml | MQL/μg/ml |
| --- | --- | --- | --- | --- |
| Al | 309.271000 | 0.974127 | 0.004980 | 0.016600 |
| As | 189.042000 | 0.999642 | 0.002725 | 0.009085 |
| Ba | 455.403000 | 0.999654 | 0.000040 | 0.000133 |
| Co | 228.616000 | 0.999621 | 0.000434 | 0.001446 |
| Cr | 283.563000 | 0.999708 | 0.000259 | 0.000864 |
| Cu | 324.754000 | 0.999696 | 0.000313 | 0.001045 |
| Fe | 259.940000 | 0.998886 | 0.000292 | 0.000974 |
| Hg | 184.950000 | 0.999334 | 0.000899 | 0.002998 |
| Li | 670.784000 | 0.999021 | 0.000022 | 0.000075 |
| Mg | 279.553000 | 0.999637 | 0.000008 | 0.000027 |
| Mn | 257.610000 | 0.999784 | 0.000046 | 0.000153 |
| Na | 588.995000 | 0.969373 | 0.000189 | 0.000628 |
| Ni | 221.647000 | 0.999616 | 0.000468 | 0.001560 |
| Pb | 220.353000 | 0.999673 | 0.002552 | 0.008507 |
| Sc | 361.384000 | 0.999662 | 0.000049 | 0.000164 |
| Sr | 407.771000 | 0.999713 | 0.000013 | 0.000042 |
| Zn | 213.856000 | 0.998290 | 0.000144 | 0.000480 |

Table s4b Trace element results for five different types of tea (μg/g, n = 6, CV% < 0.2%)

| Sample | Duyun Maojian tea | Liping fragrant tea | Shuicheng spring tea | Huaxi jasmine tea | Zunyi black tea |
| --- | --- | --- | --- | --- | --- |
| Al | 280.4852±0.0086 | 311.8826±0.0080 | 439.7463±0.0020 | 693.3524±0.0120 | 185.4244±0.0009 |
| As | 0.5793±0.0000 | 0.1790±0.0014 | — | — | — |
| Ba | 9.6669±0.0001 | 9.0551±0.0001 | 15.6448±0.0000 | 11.6869±0.0005 | 10.4797±0.0001 |
| Co | 0.2534±0.0000 | 0.3579±0.0001 | 1.1628±0.0000 | 0.4289±0.0000 | 0.2583±0.0001 |
| Cr | 1.8827±0.0003 | 2.1117±0.0001 | 1.0218±0.0001 | 1.5011±0.0001 | 1.2915±0.0001 |
| Cu | 44.7502±0.0009 | 34.0372±0.0011 | 58.7738± 0.0006 | 33.2380±0.0002 | 34.7232±0.0003 |
| Fe | 202.1361±0.0069 | 214.3164±0.0028 | 167.8999±0.0011 | 248.1415±0.0010 | 235.0554±0.0010 |
| Hg | — | — | — | — | — |
| Li | 0.1448±0.0000 | 0.1432±0.0000 | 0.0705 ±0.0000 | 0.1787±0.0000 | 0.1107±0.0000 |
| Mg | — | 1478.8833±0.0030 | — | 1637.2409±0.0440 | 1212.9151±0.0120 |
| Mn | 548.5156±0.0020 | 550.1074±0.0070 | 793.5166±0.0090 | 918.5132±0.0340 | 398.1550±0.0010 |
| Na | 289.5366±0.0058 | 254.4381±0.0029 | 358.7033±0.0150 | 293.2452±0.0641 | 343.2472±0.0156 |
| Ni | 12.3461±0.0000 | 7.6593±0.0002 | 15.8562±0.0001 | 10.0429±0.0002 | 7.0480±0.0000 |
| Pb | 2.4982±0.0006 | 1.2527±0.0015 | 1.6209±0.0005 | 1.9657±0.0005 | 0.9963±0.0000 |
| Sc | — | — | — | — | — |
| Sr | 3.3309±0.0001 | 3.0780±0.0001 | 5.6378±0.0000 | 5.3610±0.0000 | 3.2472±0.0000 |
| Zn | 319.9855±0.0034 | 248.7115±0.0067 | 299.2600±0.0024 | 194.4961±0.0015 | 157.0111±0.0016 |

Table s4c Characteristic roots of gray correlation coefficients for 15 elements

| Gray principal component | Characteristic root | Contribution rate% | Cumulative contribution rate % |
| --- | --- | --- | --- |
| 1 | 7.152 | 47.679 | 47.679 |
| 2 | 4.043 | 26.954 | 74.632 |
| 3 | 2.931 | 19.537 | 94.170 |
| 4 | 0.875 | 5.830 | 100.000 |
| … | … | … | … |

Table s4d Gray principal component vectors of 15 trace elements in 5 kinds of teas

| Element | Gray principal component vector 1 | Gray principal component vector 2 | Gray principal component vector 3 |
| --- | --- | --- | --- |
| Al | -0.093 | 0.993 | -0.077 |
| As | -0.156 | -0.284 | 0.903 |
| Ba | 0.911 | 0.377 | -0.162 |
| Co | 0.936 | 0.233 | -0.063 |
| Cr | -0.699 | -0.250 | 0.486 |
| Cu | 0.958 | 0.024 | 0.280 |
| Fe | -0.802 | 0.269 | -0.424 |
| Li | -0.824 | 0.525 | 0.205 |
| Mg | -0.728 | 0.349 | -0.545 |
| Mn | 0.208 | 0.974 | 0.009 |
| Na | 0.802 | -0.070 | -0.413 |
| Ni | 0.874 | 0.269 | 0.405 |
| Pb | -0.040 | 0.307 | 0.884 |
| Sr | 0.585 | 0.801 | -0.129 |
| Zn | 0.467 | -0.097 | 0.858 |

Table s4e Gray principal component scores and composite scores of 15 trace elements in 5 kinds of teas

| Sample | f_1_ | f_2_ | f_3_ | f | Ranking |
| --- | --- | --- | --- | --- | --- |
| Duyun Maojian tea | -0.0951 | -0.8448 | 0.3299 | -0.2086 | 3 |
| Liping fragrant tea | -0.7448 | -0.4792 | 0.2356 | -0.4382 | 4 |
| Shuicheng spring tea | 1.7377 | 0.5436 | -1.3237 | 0.7164 | 1 |
| Huaxi jasmine tea | -0.5320 | 1.4985 | 1.3267 | 0.4094 | 2 |
| Zunyi black tea | -0.3657 | -0.7181 | -0.5685 | -0.4790 | 5 |

Table s5a Determination of amino acids in five kinds of tea leaves

| Amino acid | Duyun Maojian tea | Liping Fragrant tea | Shuicheng spring tea | Huaxi jasmine tea | Zunyi Black tea |
| --- | --- | --- | --- | --- | --- |
| CySO_3_H | 99.6027±0.4194 | 110.1973±0.2189 | 186.4863±2.0334 | 147.663±0.4757 | 93.388±1.3385 |
| Asp | 2321.932±0.2466 | 2661.8703±0.397 | 3439.06±1.0096 | 2207.6303±0.6094 | 136.061±0.5692 |
| MetSON | - | - | - | - | - |
| Thr | 212.3943±0.4531 | 440.0073±0.4677 | 460.798±1.076 | 322.665±0.8428 | 8.39±0.0853 |
| Ser | 5194.7197±0.8285 | 6477.1667±0.3629 | 13757.6673±0.6369 | 7595.1427±1.1761 | 385.7627±0.6041 |
| Glu | 1966.4807±1.4068 | 2879.571±0.099 | 2698.9563±0.6412 | 2605.8843±0.6672 | 54.1733±0.1774 |
| Gly | 7.642±0.0548 | 8.101±0.0855 | - | 13.1077±0.1611 | 4.237±0.0308 |
| Ala | 182.4817±1.6622 | 172.5277±0.4369 | 295.7237±0.7511 | 257.721±0.7632 | 23.3483±0.2961 |
| (Cys)_2_ | 91.572±1.2966 | 41.756±0.4797 | 75.086±0.7965 | 24.6897±0.37 | 12.6967±0.1791 |
| Val | 497.2023±0.4486 | 318.0183±0.9878 | 995.6473±1.1979 | 241.8677±0.772 | 31.9327±0.3669 |
| Met | 190.222±1.6214 | 197.4353±1.4041 | 612.6963±1.4915 | 203.8673±1.3976 | 236.8927±0.6502 |
| Ile | 159.3447±0.3841 | 225.8433±0.1495 | 391.3417±0.5032 | 215.8513±0.2016 | 24.1963±0.4023 |
| Leu | 188.5513±0.4153 | 270.7353±0.1256 | 1338.211±0.5785 | 261.4497±1.6253 | 127.3607±1.1943 |
| Tyr | 258.239±0.1745 | 363.3773±0.3528 | 472.1507±0.4734 | 320.219±0.8244 | 147.446±0.9709 |
| Phe | 569.07±0.2063 | 682.6003±0.6751 | 936.757±0.8428 | 482.983±0.6067 | 539.8313±1.2651 |
| His | 143.2407±0.2991 | - | 165.1473±1.0453 | 67.9307±0.5539 | 21.7163±0.3252 |
| Lys | 26.2397±0.1702 | 20.495±0.144 | 676.3767±1.1687 | 258.3797±0.9495 | 35.272±0.3092 |
| NH_4_ | 122.5603±0.1695 | 58.499±0.3694 | 125.8057±1.707 | 138.7137±1.064 | 63.884±0.1741 |
| Arg | 252.9137±0.2877 | 180.3397±0.8711 | 1367.7227±2.0927 | 478.5573±1.377 | 1048.308±0.0803 |
| Pro | 29.6217±0.0896 | 37.4737±0.2261 | 255.766±0.8428 | 19.213±0.1273 | 12.5303±0.1786 |

Table s5b Characteristic roots of gray correlation coefficients for the 19 amino acids in 5 teas

| Gray factors | Characteristic root | Contribution rate% | Cumulative contribution rate % |
| --- | --- | --- | --- |
| 1 | 11.495 | 60.500 | 60.500 |
| 2 | 4.407 | 23.194 | 83.694 |
| 3 | 1.670 | 8.788 | 92.482 |
| 4 | 1.428 | 7.518 | 100.000 |
| … | … | … | … |

Table s5c The gray factor loading matrix for the 19 amino acids in 5 teas

| Amino acids | Gray factor loading matrix 1 | Gray factor loading matrix 2 | Gray factor loading matrix 3 | Gray factor loading matrix 4 |
| --- | --- | --- | --- | --- |
| CySO_3_H | 0.012 | 0.952 | 0.147 | -0.267 |
| Asp | 0.839 | 0.147 | 0.475 | 0.222 |
| Thr | 0.720 | 0.121 | 0.669 | -0.140 |
| Ser | -0.349 | 0.701 | 0.621 | 0.040 |
| Glu | 0.490 | 0.405 | 0.772 | -0.004 |
| Gly | -0.481 | 0.843 | 0.223 | -0.091 |
| Ala | 0.798 | 0.562 | 0.178 | 0.127 |
| (Cys)_2_ | 0.351 | -0.129 | 0.109 | 0.921 |
| Val | 0.970 | -0.076 | -0.018 | 0.232 |
| Met | 0.975 | -0.123 | -0.182 | -0.024 |
| Ile | 0.974 | 0.118 | 0.193 | 0.019 |
| Leu | 0.991 | -0.079 | -0.106 | 0.001 |
| Tyr | 0.916 | 0.127 | 0.377 | -0.044 |
| Phe | 0.947 | -0.297 | 0.122 | -0.008 |
| His | 0.729 | 0.048 | -0.206 | 0.652 |
| Lys | 0.984 | 0.051 | -0.165 | -0.033 |
| NH_4_ | 0.384 | 0.786 | -0.207 | 0.437 |
| Arg | 0.754 | -0.256 | -0.557 | -0.235 |
| Pro | 0.987 | -0.110 | -0.114 | 0.018 |

Table s5d Gray factor scores and composite gray factor scores for the 19 amino acids in the five kinds of tea

| Sample | F_1_ | F_2_ | F_3_ | F_4_ | F | Ranking |
| --- | --- | --- | --- | --- | --- | --- |
| Duyun Maojian tea | -0.4815 | -0.1119 | -0.0002 | 1.7192 | -0.1880 | 3 |
| Liping fragrant tea | -0.2780 | -0.5501 | 1.5764 | -0.5788 | -0.2007 | 4 |
| Shuicheng spring tea | 1.7604 | -0.1868 | -0.2565 | 0.0155 | 1.0004 | 1 |
| Huaxi jasmine tea | -0.2873 | 1.7069 | -0.1222 | -0.4347 | 0.1786 | 2 |
| Zunyi black tea | -0.7137 | -0.8582 | -1.1975 | -0.7212 | -0.7903 | 5 |
